# Laparoscopic lateral suspension combined with uterosacral ligament suspension for advanced apical pelvic organ prolapse: preliminary outcomes of a retrospective series

**DOI:** 10.3389/fmed.2026.1763296

**Published:** 2026-04-15

**Authors:** Shuxu Tian, Chenchen Geng, Xuan Liu, Juan Hao, Huangbei Song, Shuping Zhao

**Affiliations:** 1 Department of Gynecology, Qingdao Women and Children’s Hospital, Qingdao University, Qingdao, Shandong, China; 2 Department of Ultrasound, Qilu Hospital of Shandong University (Qingdao), Qingdao, Shandong, China

**Keywords:** apical prolapse, laparoscopic lateral suspension, pelvic organ prolapse, uterine preservation, uterosacral ligament suspension

## Abstract

**Background:**

Laparoscopic lateral suspension (LLS) is a novel technique for repairing anterior and apical pelvic organ prolapse (POP). However, the efficacy is limited for advanced apical defects, particularly when combined with posterior compartment prolapse. This preliminary study evaluated the short-term outcomes of mesh-based LLS combined with uterosacral ligament suspension (USLS) for treating advanced apical prolapse with involvement of both the anterior and posterior compartments.

**Methods:**

Between January 2022 and June 2024, 28 women with apical POP ≥ stage III underwent mesh-based LLS with USLS. Titanized polypropylene mesh was placed on the vesicovaginal fascia, and the posterior compartment defects were addressed with USLS. All patients preserved their uterus. Clinical outcomes were assessed using the gynecological examination and the pelvic organ prolapse quantification (POP-Q) system. Objective anatomic success was defined as POP-Q stage <II at all points at 12-month follow-up. Subjective outcomes were measured with the Pelvic Floor Distress Inventory Questionnaire.

**Results:**

The mean age of participants was 57.71 ± 10.36 years, with a mean BMI of 25.48 ± 2.87 kg/m^2^. Concomitant procedures included distal posterior vaginal wall repair (*n* = 16), partial cervical resection (*n* = 5), hysteroscopy (*n* = 9), and other procedures. No serious perioperative complications occurred in the patients. While anatomical restoration was high in this small cohort (100%), these results are preliminary. Subgroup analysis showed no significant difference in point Bp restoration between patients with and without distal vaginal repair (*p* > 0.05). No serious complications or mesh exposure occurred.

**Conclusion:**

Combining LLS with USLS is a safe and potentially effective treatment for advanced, multi-compartment apical prolapse in this preliminary cohort. While short-term outcomes are encouraging, the lack of a control group and the potential confounding effect of distal vaginal repair necessitate larger studies to confirm long-term durability.

## 1 Introduction

Pelvic organ prolapse (POP) is common among women, and advanced apical prolapse poses particular challenges for durable, effective repair. Sacrocolpopexy (SCP) has long been regarded as the gold standard for apical prolapse, offering high anatomic success but requiring advanced technical expertise and carrying risks such as hemorrhage and mesh-related complications (1, 2). Laparoscopic lateral suspension (LLS) has emerged as a minimally invasive alternative that provides robust anterior and apical support with less perioperative complications and facilitates uterine preservation (3, 4). However, LLS alone may not adequately address the posterior compartment, particularly in the presence of an enterocele or significant posterior vaginal wall prolapse (3–7). Consequently, combining complementary suspension techniques has gained attention to correct multicompartment defects and improve outcomes (8). Integrating LLS for anterior/apical support with uterosacral ligament suspension (USLS) for posterior reinforcement may provide comprehensive correction for advanced apical prolapse while minimizing mesh exposure and perioperative risk. In this retrospective series, the preliminary clinical outcomes in 28 patients with advanced apical POP treated with combined LLS and USLS are reported.

## 2 Methods

This single-center retrospective observational series was conducted at the Qingdao Women and Children’s Hospital with the Institutional Ethics Committee approval (Approval No. QFELL-YJ-2024-137). All participants provided written informed consent. From January 2022 to June 2024, 28 women with symptomatic severe apical POP (POP-Q stage ≥III) and significantly reduced quality of life (assessed by the Pelvic Floor Distress Inventory-Short Form 20, PFDI-20) underwent LLS combined with USLS procedures. Standardized preoperative evaluations used the POP-Q system. Two gynecologic surgeons trained in both procedures performed all operations.

Inclusion criteria included (1) female patients with apical compartment POP (POP-Q stages III–IV) and concomitant anterior and posterior compartment defects, (2) symptomatic prolapse requiring surgical intervention, (3) absence of serious systemic disease and suitability for surgery, (4) complete baseline and postoperative follow-up data, and (5) willingness to participate. Exclusion criteria included (1) history of gynecological cancer, (2) prior pelvic floor reconstruction at the same anatomical site, (3) medical comorbidities contraindicating surgery, and (4) refusal to use mesh.

Preoperative assessment included the gynecologic examination, liquid-based cervical cytology, and human papillomavirus testing. All patients underwent a clinical cough stress test; in those with stages III–IV prolapse, this was performed after manual reduction of the prolapse to identify occult stress urinary incontinence (SUI). Collected data encompassed demographics (age, BMI, and obstetric and surgical history), the prolapse type, and the POP-Q scores (preoperatively and postoperatively); operative details (approach, time); estimated blood loss; intra- and postoperative complications; and other perioperative parameters. Follow-up visits were scheduled at 1, 3, 6, and 12 months and annually thereafter. At each visit, a standardized follow-up form was used to record: (1) anatomical success through POP-Q measurements; (2) the presence or absence of mesh exposure or vaginal erosions by speculum examination; and (3) the emergence of any new-onset symptoms, including pelvic pain, dyspareunia, or urinary/bowel dysfunction. All patients completed at least 12-month follow-up. Subjective outcomes were assessed using the PFDI-20 at the 12-month visit.

### 2.1 Surgical procedure

LLS was performed as described in Dubuisson et al. (5). POP was corrected using a T-shaped (TiLOOP^®^) prosthesis attached to the cervix and anterior vaginal wall. The mesh was sutured to the cervix with three to four non-absorbable sutures and to the anterior vaginal wall with six to eight absorbable sutures. The mesh arms were then routed laterally toward the abdominal wall.

For USLS, the uterosacral ligament and the ureter were identified. The midportion of the uterosacral ligament was sutured in a spiral fashion to the posterior cervix using non-absorbable sutures. The cervical fascia ring was reinforced, and the cervix was further elevated. When a higher suspension was required, the peritoneum overlying the uterosacral ligament was incised from the cervix toward the rectal reflection with the ureter kept lateral, and the ligament was tensioned appropriately. To mitigate the risk of ureteral injury, the peritoneum overlying the uterosacral ligament was incised from the posterior cervix toward the sacral reflection. This facilitated direct anatomical visualization and lateralization (lateral displacement) of the ureter, separating it from the ligament to create a safe zone for suture placement. Ureteral patency was confirmed intraoperatively through cystoscopy.

Uterine preservation was planned for all patients. Partial cervical resection was performed for marked cervical elongation. When significant posterior vaginal wall laxity or distal bulging persisted after suspension, a distal posterior vaginal repair was additionally performed. This procedure was limited to DeLancey level III (perineorrhaphy and repair of the lower one-third of the vagina) to address the introital relaxation. A full-length posterior colporrhaphy was not performed in any patients, ensuring that the restoration of levels I and II posterior support was attributable to the USLS. Postmenopausal women were advised to undergo concurrent salpingo-oophorectomy.

### 2.2 Outcome and analysis

The primary outcome was objective anatomic improvement, defined as POP-Q stage <II at all follow-up points. Secondary outcomes included quality of life assessed with the PFDI-20 and its subscales (Pelvic Organ Prolapse Distress Inventory-6, POPDI-6; Colorectal-Anal Distress Inventory-8, CRADI-8; and Urinary Distress Inventory-6, UDI-6) preoperatively and at 12 months and mesh-related complications. Descriptive statistics (mean ± SD) characterized the cohort; estimated blood loss was summarized as median (first quartile and third quartile). Pre-post comparisons used paired *t*-tests or Wilcoxon signed-rank tests according to data distribution (SPSS version 26.0), with a two-sided alpha of 0.05.

## 3 Results

Between January 2022 and June 2024, 28 patients underwent POP repair with combined LLS and USLS. The mean age was 57.71 ± 10.36 years (range, 34–74), and the mean BMI was 25.48 ± 2.87 kg/m^2^ (range, 18.02–33.30). Postmenopausal women comprised 71.43% of the cohort. Hypertension was present in 10 patients, diabetes in 1, and hypothyroidism in 2. Other gynecologic conditions included endometrial polyps/hyperplasia (*n* = 9), uterine leiomyomas (*n* = 6), and ovarian cysts (*n* = 1).

All patients had severe apical prolapse (POP-Q stage ≥III) with varying degrees of anterior and posterior involvement. Mild to moderate stress urinary incontinence was reported in 12 patients; dysuria and constipation were common prolapse-related symptoms. Uterine preservation was achieved in all patients. The mean operative time was 146.57 ± 32.40 min (range, 90–205). Median estimated blood loss was 43.93 mL (10, 50), with higher losses in those undergoing concomitant myomectomy. The average hospital stay was 6.39 ± 1.45 days (range, 4–9), reflecting local institutional protocols for postoperative monitoring. Concomitant procedures included posterior vaginal wall repair (*n* = 16), partial cervical resection (*n* = 5), hysteroscopy (*n* = 9), bilateral salpingo-oophorectomy (*n* = 12), myomectomy (*n* = 6), and ovarian cystectomy (*n* = 1) (see Tables 1–3).

**Table 1:** Characteristics of the study population (*n* = 28).

Characteristics	Value
Age (year), mean ± SD (range)	57.71 ± 10.36 (34–74)
Height (cm), mean ± SD (range)	159.68 ± 5.46 (156–173)
Weight (kg), mean ± SD (range)	65.03 ± 8.36 (45–83)
BMI (kg/m^2^), mean ± SD (range)	25.48 ± 2.87 (18.02–33.30)
Parity, mean ± SD (range)	1.64 ± 0.67 (1–3)
Menopause, *n* (%)	20 (71.43%)
Comorbidity, *n* (%)	
Hypertension and cardiovascular disease	10
Diabetes	1
Hypothyroidism	2
Previous POP surgery, *n*	0
Previous hysterectomy, *n*	0
Previous UI surgery, *n*	1

BMI, body mass index; POP-Q, pelvic organ prolapse; UI, urinary incontinence.

**Table 2:** Preoperative pelvic floor characteristics of the study population.

POP-Q stage, *n* (%)	Value
Apical prolapse	
Stage III	13 (46.43%)
Stage IV	15 (53.57%)
Anterior prolapse	
Stage I	0
Stage II	11 (39.29%)
Stage III	5 (17.86)
Stage IV	12 (42.86%)
Posterior prolapse	
Stage I	3 (10.71%)
Stage II	12 (42.86%)
Stage III	6 (21.43%)
Stage IV	7 (25.00%)
Mild–moderate SUI	12 (42.86%)
Dysuria	3 (10.71%)

POP-Q, pelvic organ prolapse quantification; SUI, stress urinary incontinence.

**Table 3:** Perioperative and complication characteristics (*n* = 28).

Characteristic	Value
Operative time (min), mean ± SD (range)	146.57 ± 32.40 (90–205)
Concomitant procedures^a^, *n* (%)	
Posterior vaginal wall repair	16 (57.14%)
Partial cervical resection	5 (17.86%)
Bilateral salpingo-oophorectomy	12 (42.86%)
Hysteroscopy	9 (32.24%)
Myomectomy	6 (21.43%)
Ovarian cystectomy	1 (3.57%)
Estimated blood loss (mL), median (first quartile, third quartile)	43.93 (10, 50)
Hospital stays (day), mean ± SD (range)	6.39 ± 1.45 (4–9)
Intraoperative complication, *n* (%)	0
Blood transfusion, *n* (%)	0
Conversion to laparotomy, *n* (%)	0
Bladder injury, *n* (%)	0
Bowel injury during laparoscopy, *n* (%)	0
Mesh erosion/ exposure, *n* (%)	0

a One patient may have more than one associated procedure.

All patients completed at least 12-months follow-up. Objective anatomic criteria were met in every patient (100%), and no recurrences were observed. The PFDI-20, POPDI-6, CRADI-8, and UDI-6 scores improved significantly after 12 months (Table 4). Subgroup analysis showed no significant difference in point Bp restoration between patients with and without distal vaginal repair (*p* > 0.05), suggesting that upper posterior wall support was primarily restored by the USLS. No mesh exposure or serious intraoperative complications occurred. Postoperative issues were limited to mild lower abdominal pain in some patients. One patient developed position-dependent urinary incontinence at 1 month, which improved with pelvic floor rehabilitation.

**Table 4:** Comparison of PFDI-20 at preoperative and postoperative in 12-month follow-up visit.

Questionnaire	Preoperative (mean ± SD)	Postoperative (mean ± SD)	*p*-value
PFDI-20	100.41 ± 23.41	42.11 ± 11.94	*p* < 0.01
POPDI-6	53.72 ± 16.27	14.29 ± 5.27	*p* < 0.01
CRADI-8	22.88 ± 10.96	13.40 ± 6.01	*p* < 0.01
UDI-6	23.81 ± 11.07	14.43 ± 5.16	*p* < 0.01

CRADI-8, Colorectal-Anal Distress Inventory-8; PFDI-20, Pelvic Floor Distress Inventory-Short Form-20; UDI-6 Urinary Distress Inventory-6.

## 4 Discussion

Complex, high-grade apical POP remains a surgical challenge. While SCP is widely regarded as the gold standard for apical prolapse (1, 9), advances in laparoscopy have enabled effective alternatives. LLS demonstrates high efficacy for anterior and middle compartment defects but may provide insufficient posterior support, especially in patients with enterocele or substantial posterior vaginal wall prolapse (3–7). These limitations support combining LLS with complementary techniques. By reattaching the apex to the uterosacral ligaments, USLS augments posterior support and mitigates posterior vaginal wall descent and bowel prolapse (10). By incorporating USLS, we utilized native tissue to reinforce the posterior vault, thereby avoiding the use of mesh in the rectovaginal septum. Together, LLS and USLS offer compartment-spanning support, addressing the full spectrum of defects in complex patients.

In this cohort of 28 women with complex, high-grade apical POP, combined LLS and USLS yielded a high objective anatomic success rate (100% at 12 months). However, this pilot study represents preliminary findings, and these results must be interpreted with caution, as anatomical restoration rates often fluctuate over long observation periods.

While SCP achieves high long-term success for apical prolapse (approximately 90%) (2, 11, 12), it carries risks related to sacral promontory dissection, including vascular and nerve injury and rare spondylodiscitis. Posterior mesh use in SCP increases erosion risk, reported at up to 3–10.5% (2, 11, 13). The LLS + USLS approach offers an alternative that avoids the sacral promontory, thus eliminating the risk of presacral vascular injury, and allows uterine preservation. Reducing posterior mesh placement is important because mesh in the rectovaginal septum increases erosion risk (14). Notably, while SCP is criticized for presacral risks, USLS is historically associated with ureteral injury or entrapment. To ensure safety, the protocol utilized a visual-dissection approach through peritoneal incision and ureteral lateralization. By anatomically isolating the ureter from the suspension site, we minimized the risk of iatrogenic entrapment. This proactive dissection, confirmed by cystoscopy, is essential for maintaining a high safety profile. The study observed no mesh exposures or serious complications, supporting the safety of this mesh-sparing combined strategy for posterior compartment defects.

The primary goal of prolapse surgery is improvement in function and symptom relief rather than perfect anatomic restoration. The results—100% anatomic success, significant improvements in PFDI-20, and subscale scores in the short-term—are consistent with this goal. Recent studies suggest that tailored or staged repairs based on defect distribution can prevent overtreatment and reduce complications (8). The anatomic success observed in this study aligns with short- and medium-term outcomes reported for SCP and LLS (3, 14, 15), and the quality-of-life gains further support the effectiveness of the combined technique. This study, which combined the LLS + USLS approach, adds to the evidence supporting tailored, mesh-sparing strategies for complex POP.

A critical distinction in this study is the management of the posterior compartment. While 16 patients underwent concomitant vaginal repair, these were strictly restricted to the distal level III compartment (perineorrhaphy) to address introital relaxation. Successful treatment of deep posterior defects (rectocele and enterocele) was achieved by the apical traction provided by the USLS. This sequential approach ensures that levels I and II support is restored laparoscopically, while vaginal surgery is reserved for distal functional outlet repair.

Both LLS and USLS are well-suited to minimally invasive laparoscopy, with relatively short learning curves (approximately 10–15 patients) (5, 10, 15). Uterine preservation may reduce operative time and blood loss, align with patient preferences, obviate morcellation, and may decrease the risk of subsequent posterior compartment prolapse (8). Furthermore, the results should be considered alongside other emerging techniques such as pectopexy, which also avoids the presacral space but utilizes different anchoring points (16).

This study has several significant limitations. First, the retrospective design without a control group introduces selection bias. Second, the small sample size (*n* = 28) is insufficient to detect rare complications. Third, the 12-month follow-up is a short-term interval for pelvic floor reconstruction. Fourth, there is a significant degree of procedural heterogeneity within the cohort. A high proportion of patients underwent concomitant surgeries, including salpingo-oophorectomy, myomectomy, and partial cervical resection. While these procedures are often necessary in real-world clinical practice for women with multicompartment POP and coexisting gynecological pathology, they act as confounding variables. Specifically, these concomitant procedures may have influenced perioperative data—such as operative time and blood loss—and may have impacted subjective patient-reported outcomes (PFDI-20) independently of the prolapse repair itself. Consequently, this heterogeneity limits the ability to isolate the specific anatomical and functional contributions of the combined LLS + USLS technique. Finally, the inclusion of distal vaginal repairs remains a potential confounding factor in subjective outcomes.

## 5 Conclusion

In this preliminary retrospective series, combining LLS with USLS was highly effective for short-term anatomical restoration of apical POP. This approach integrates the strengths of LLS and USLS while using a visual-dissection technique to ensure ureteral safety. However, larger prospective trials are required to confirm the long-term utility of this combined approach compared with the existing gold standard.

## Data Availability

The original contributions presented in the study are included in the article/supplementary material, further inquiries can be directed to the corresponding author.
